# Beyond an inflammatory mediator: Interleukin‐1 in neurophysiology

**DOI:** 10.1113/EP090780

**Published:** 2023-04-09

**Authors:** Tatiane S. Lima

**Affiliations:** ^1^ Department of Biological Sciences California State Polytechnic University Pomona California USA

**Keywords:** central nervous system, cytokine, inflammation, interleukin‐1β, neurophysiology

## Abstract

Since its discovery in the early 1940s, the interleukin‐1 (IL‐1) cytokine family has been associated primarily with acute and chronic inflammation. The family member IL‐1β is produced by different leucocytes, endothelial cells and epithelial cells. This cytokine has been characterized as a key modulator of inflammation and innate immunity because it induces the transcription of several downstream inflammatory genes. More recently, several groups have demonstrated that IL‐1β production is also required to maintain homeostasis in several organ systems. This review focuses on providing an overview of the more recently characterized role of IL‐1β in the physiology of the CNS. So far, IL‐1β signalling has been implicated in neuronal survival, neurite growth, synaptic pruning, synaptic transmission, neuroplasticity and neuroendocrine functions.

## Abstract (highlights)

- **What is the topic of this review?**
  This review focuses on the physiological role of the cytokine interleukin‐1β in the CNS.
- **What advances does it highlight?**
  Traditionally, interleukin‐1β is known as a key mediator of inflammation and immunity. This review highlights the more recent findings describing how interleukin‐1β signalling is required to maintain homeostasis in the CNS.

## 1 INTRODUCTION

The initial studies that led to the discovery of interleukin 1 (IL‐1) date from the early 1940s, when Eli Menkin reported a protein present in the supernatant of rabbit leucocytes that could induce rapid onset of fever (Atkins, 1984). Subsequent studies showed that human leucocytes produce a similar protein (Dinarello et al., 1977). When the IL‐1 complementary DNA was cloned, the recombinant protein was also pyrogenic in humans (Auron et al., 1984; Ogilvie et al., 1996). Over the past two decades, several other cytokines of the IL‐1 family have been identified, and to date, the family comprises 11 members (Sims & Smith, 2010).

The IL‐1 cytokine family is associated primarily with acute and chronic inflammation. Interleukin‐1β (IL‐1β) is the best‐characterized cytokine from this family. It can be produced by different leucocytes, endothelial cells and epithelial cells. This cytokine is a major mediator of the innate immune response and several autoinflammatory diseases characterized by fever, rash and arthritis. During the last 25 years, several IL‐1 inhibitors have been developed, many of them are currently in clinical trials, and a few are already available for clinical use (Gabay et al., 2010). Anakinra was the first IL‐1β antagonist approved for clinical use (Kary & Burmester, 2003). It was approved in the USA in 2001 and in the EU in 2002. This is an interleukin‐1 receptor antagonist (IL‐1Ra) used in the treatment of rheumatoid arthritis, neonatal‐onset multisystem inflammatory disease and deficiency of interleukin‐1 receptor antagonist (Cvetkovic & Keating, 2002). In 2021 (Europe) and 2022 (USA), the use of anakinra was authorized to treat coronavirus disease 2019 patients with pneumonia at risk of progressing to severe respiratory failure (Khani et al., 2022). Besides anakinra, rilonacept and canakinumab are also IL‐1 inhibitors available for clinical use. Rilonacept blocks IL‐1β signalling by acting as a soluble decoy receptor, and canakinumab is an anti‐IL‐1β monoclonal antibody (Dinarello et al., 2012).

In addition to the pro‐inflammatory effect of IL‐1 and its importance in response to infections, recent studies suggest that tightly regulated IL‐1β production is required to maintain homeostasis in several organ systems. This review provides an overview of the more recently characterized role of IL‐1β in the physiology of the CNS, one of the systems with the strongest evidence for a role of IL‐1β in homeostasis, and for which some of the molecular mechanisms have already been explored. The review discusses recent findings describing new functions of a cytokine traditionally known for its role as the master regulator of immunity and inflammation and highlights the complexity and effectiveness of the tightly regulated human body in health and disease.

## 2 INTERLEUKIN‐1β SYNTHESIS, PROCESSING AND RELEASE

**FIGURE 1:**
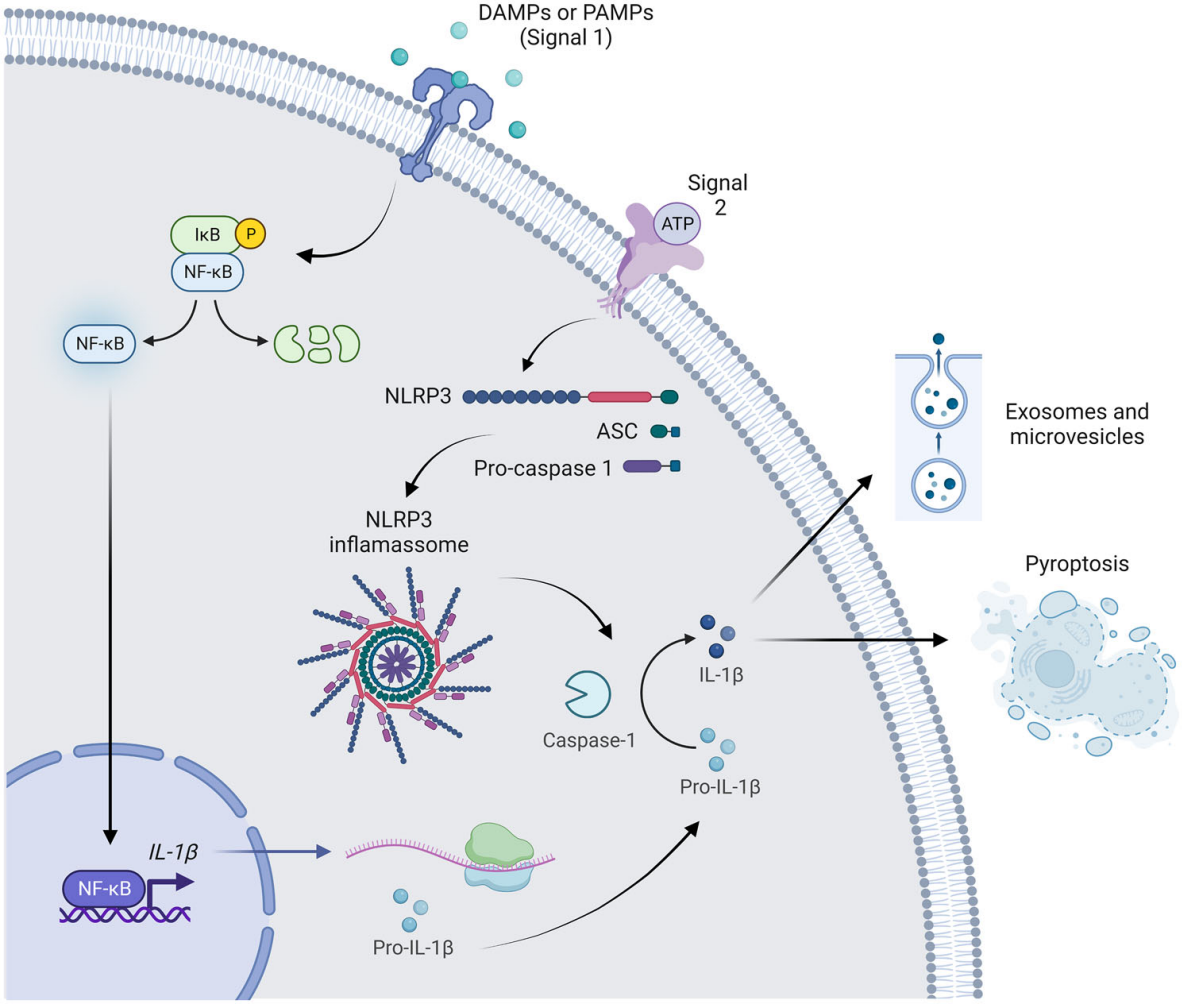
Interleukin‐1β processing. Interleukin‐1β transcription is induced by PAMPs or DAMPs through activation of the NF‐κB signalling pathway (signal 1). The protein is produced as an inactive peptide (pro‐IL‐1β) that requires processing. A second stimulatory signal, such as ATP or ROS, induces assembly and activation of the inflammasome in the cytosol. Upon inflammasome activation, the protease pro‐caspase 1 is cleaved into caspase 1, which, in turn, cleaves pro‐IL‐1β into mature IL‐1β. Active IL‐1β can be released through pyroptosis or the release of exosomes and/or microvesicles. Created with BioRender. Abbreviations: ASC, adaptor molecule apoptosis‐associated speck‐like protein containing a CARD; ATP, adenosine trifosfate; DAMPs, damage‐associated molecular patterns; IκB, inhibitor of κB; IL‐1β, interleukin‐1β; NF‐κB, nuclear factor kappa B; NLRP3, NLR family pyrin domain containing 3; PAMPs, pathogen‐associated molecular patterns; ROS, reactive oxygen species.

Although IL‐1β is produced by different cell types, most of the studies that determined the pathways involved in its synthesis, processing and release were characterized in cells of the innate immune system, such as monocytes and macrophages. Interleukin‐1β is synthesized as a precursor peptide (pro‐IL‐1β, 31 kDa) that requires cleavage to generate the mature active form of IL‐1β (17 kDa). Transcription and translation of the pro‐form were traditionally described as being induced in response to pathogen‐associated molecular patterns (PAMPs) (Martinon et al., 2009; Meylan et al., 2006) and damage‐associated molecular patterns (DAMPs) (Dostert et al., 2008; Halle et al., 2008) through activation of the nuclear factor kappa B (NF‐κB) signalling pathway (Figure 1).

Processing of pro‐IL‐1β into its active form is dependent on the assembly and activation of a multi‐protein complex known as the inflammasome, and the subsequent activation of the protease caspase 1 (Weber et al., 2010). The NLRP3 inflammasome is the best characterized so far (Hoffman et al., 2001). This complex is formed by a cytosolic sensor with a sensing C‐terminal leucine‐rich repeat domain, a central nucleotide‐binding domain and an N‐terminal pyrin domain (Schroder & Tschopp, 2010). During activation, the N‐terminal domain recruits an adaptor protein, the apoptosis associated speck‐like protein, containing a caspase‐recruitment domain (ASC). The precursor pro‐caspase 1 is then recruited to ASC and activated. The mature caspase 1 cleaves pro‐IL‐1β into the mature IL‐1β (Thornberry et al., 1992). Besides caspase 1 and caspase 8, elastase can also cleave pro‐IL‐1β (Pyrillou et al., 2020). This cytokine lacks leader peptides and therefore it cannot be secreted through the conventional pathway dependent on the endoplasmic reticulum and the Golgi apparatus.

Despite intense efforts, the mechanism of IL‐1β secretion is not completely understood. Several observations point to the existence of multiple mechanisms that could be induced upon different stimuli and result in different amounts of IL‐1β being released. Some of the mechanisms demonstrated so far are pyroptosis, a lytic form of cell death that depends on the pore‐forming protein gasdermin‐D (Shi et al., 2017), and release through exosomes and microvesicles, which allows the cell to remain alive (Qu et al., 2007).

## 3 THE TRADITIONAL ROLE OF INTERLEUKIN‐1β AS A MASTER REGULATOR OF INFLAMMATION AND INNATE IMMUNITY

**FIGURE 2:**
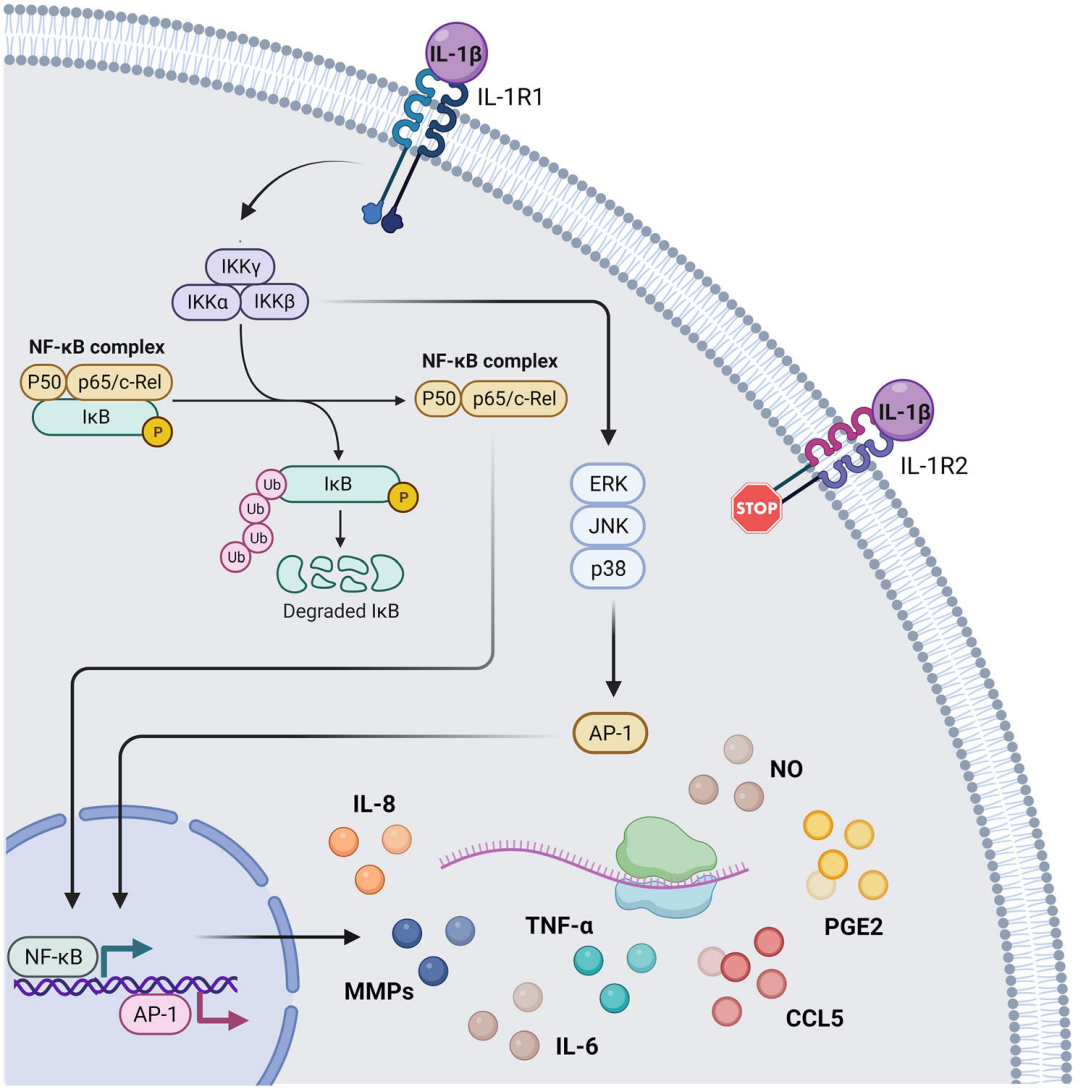
Interleukin‐1β‐induced inflammatory genes. Interleukin‐1β binding to IL‐1R1 triggers the activation of the NF‐κB and MAPK signalling pathways, both of which induce the transcription of several inflammatory genes, such as interleukin 8 (IL‐8), interleukin 6 (IL‐6), chemokine (C‐C motif) ligand 5 (CCL5), prostaglandin E2 (PGE2), tumor necrosis factor alpha (TNF‐α), nitric oxide (NO) and different metalloproteinases (MMPs). Created with BioRender. Abbreviations: AP‐1, activator protein 1; ERK, extracellular signal‐regulated kinase; IκB, inhibitor of κB; IκBα, IκB kinase alpha; IκBβ, IkB kinase beta; IkBγ, IκB kinase gamma; IL‐1β, interleukin‐1β; IL‐1R1, interleukin‐1 receptor 1; IL‐1R2, interleukin‐1 receptor 2; JNK, c‐Jun N‐terminal kinase; NF‐κB, nuclear factor kappa B.

The innate immune system has evolved to use signalling receptors that monitor the extracellular space and different subcellular compartments for signs of pathogens or damage. The inflammasome is a multi‐protein complex formed by a sensor molecule, an adaptor molecule and the protease caspase 1. Different molecules found during infection (PAMPs) or tissue damage (DAMPs) can trigger activation of the inflammasome and the processing and release of IL‐1β, a key mediator of the inflammatory response. Binding of IL‐1β to the IL‐1 receptor 1 (IL‐1R1), present in the surface of a variety of immune and endothelial cells, activates the NF‐κB and p38 mitogen‐activated protein kinase (MAPK) signalling pathways, both of which induce the transcription of several downstream inflammatory genes that contribute to the recruitment of additional immune cells to the site of injury and modulate the function of immune cells to maximize their antimicrobicidal activities and the immune response (Gabay et al., 2010). Besides binding to IL‐1R1, IL‐1β can also bind to IL‐1 receptor 2 (IL‐1R2), which acts as a decoy receptor (Colotta et al., 1994; Figure 2).

## 4 NEW ROLES OF INTERLEUKIN‐1β IN NEUROPHYSIOLOGY

For a long time, increased IL‐1β expression in the CNS and altered microglial morphology were considered hallmarks of CNS inflammation. Correlations of IL‐1β levels in the plasma or CNS and disease severity have been widely reported. Infection of the CNS and neuronal loss after ischaemic brain damage are both aggravated by IL‐1β (Huang et al., 2003).

The induction of IL‐1β expression in the CNS has also been shown in models of acute brain injury (Lu et al., 2007), Alzheimer's disease (Kitazawa et al., 2011), Parkinson's disease (Sznejder‐Pachołek et al., 2017), CNS autoimmunity (Paré et al., 2017), anxiety (Rossi et al., 2012), depression (Maes et al., 2012) and autism (Wu et al., 2017). In animal models of cerebral stroke, treatment with an IL‐1Ra reduces infarct volume by 36% (McCann et al., 2016). The same antagonist blocks stress‐induced anxiety and depression (Gentile et al., 2016) and improves clinical outcomes in models of epilepsy (Noe et al., 2013).

In addition to the potential inflammatory outcomes of IL‐1β in the CNS, several reports have also demonstrated a key physiological role for this cytokine in the brain. Moreover, recent studies have made progress on understanding the molecular mechanisms associated with this homeostatic function of IL‐1β. In the brain, IL‐1β is produced by microglia, astrocytes and endothelial cells (Davies et al., 1999; Giulian et al., 1986). In physiological conditions, IL‐1R1 is expressed by endothelial cells, ependymal cells, choroid plexus cells and dentate gyrus neurons. The receptor is also expressed at low levels by astrocytes; however, it is not expressed by microglia or brain macrophages (Liu et al., 2019).

### 4.1 Neuronal survival and neurite growth

Interleukin‐1β is highly expressed in the developing brain and directly affects the neuronal precursor cells. By using neuronal culture models, it has been demonstrated that high concentrations (500 ng/mL) of IL‐1β for extended periods of time (3–5 days) are neurotoxic; however, short‐term exposure (1 day) to low concentrations (10 ng/mL) is not; in fact, it stimulates in vitro migration of cortical neurons and promotes neurite outgrowth, suggesting a role for IL‐1β during brain development (Boato et al., 2011). Additional mechanistic studies have demonstrated that activation of the non‐canonical Wnt5a signalling (Wnt5a/RhoA/ROCK/JNK pathway) by IL‐1β is required for neuronal differentiation, especially neurite outgrowth. Interleukin‐1β increases transcript and protein levels of Wnt5a by inducing NF‐κB activation. Still, the use of Wnt5a knockdowns confirmed its requirement for IL‐1β‐induced neural differentiation. In a similar manner, differentiation of neuronal precursor cells was induced by the exogenous administration of Wnt5a (Park et al., 2018). A separate study identified p38 MAPK as an additional mediator of neurite outgrowth. The authors observed an increase in IL‐1β expression in Schwann cells after a sciatic nerve injury. Interleukin‐1β‐dependent neurite outgrowth was observed in dorsal root ganglion neurons and cerebellar granule neurons. This effect depends on inhibition of RhoA, a small GTPase, via the p38 MAPK pathway (Temporin et al., 2008). Additionally, IL‐1β stimulates neurotrophin‐3, neurogenin 1 and brain‐derived neurotrophic factor, which might help to explain how IL‐1β induces neuronal survival and neurite growth (Boato et al., 2011; Correale & Villa, 2004; Park et al., 2018).

Interleukin‐1β stimulates the survival of neurons by promoting the expression of nerve growth factors (Carlson et al., 1999). In contrast, in a model of depression induced by tumor necrosis factor alpha (TNF‐α), IL‐1β expression in the hippocampus and reduced neurogenesis in dentate gyrus were observed. These effects, together with the depression behaviours were blocked by the use of IL‐1Ra (Akama & Van Eldik, 2000). Similar effects were observed in a model of chronic mild stress (Goshen et al., 2008). Such antineurogenic and depressive effects might be explained by IL‐1β‐induced activation of the NF‐κB pathway in microglia, followed by production of NO (Ferreira et al., 2010). This is supported by evidence that NF‐κB inhibition reverts the antineurogenic and depressive effects (Song et al., 2013). Nitric oxide can be neuroprotective in low concentrations; however, in large amounts, it inhibits mitochondrial cytochrome oxidase in neurons, which causes them to depolarize and to release glutamate (Bal‐Price & Brown, 2001).

### 4.2 Neuroplasticity

Interleukin‐1β also plays a role in regulating normal sleep. Cerebrospinal fluid concentrations of IL‐1β increase during sleep in comparison to the awake state of animals (Taishi et al., 1998). Its brain expression follows a diurnal rhythm, with increasing levels of IL‐1β being associated with increased sleepiness, whereas inhibition of IL‐1β signalling reduces sleep. Besides IL‐1β, TNF‐α, growth hormone‐releasing hormone and NO are also involved in sleep regulation. The increase in NO levels might be induced by IL‐1β (Krueger et al., 1998). The mechanisms associated with this effect are partly understood and are associated with interactions of IL‐1β with classical neurotransmitters, such as acetylcholine, glutamate, adenosine and monoamines (Imeri & Opp, 2009). Low concentrations of IL‐1β in the dorsal raphe nuclei promote non‐rapid eye movement sleep by enhancing the inhibitory effects of GABA and reducing the firing rate of wake‐active serotonergic neurons. In a different region, the hypothalamic preoptic area/basal forebrain region, IL‐1 stimulates serotonin release from axon terminals, which, in turn, inhibits cholinergic neurons involved in cortical activation and stimulates the synthesis of IL‐1β. The newly synthesized IL‐1β inhibits wake‐promoting neurons and activates a subset of sleep‐promoting neurons in the preoptic area/basal forebrain. Interleukin‐1β in the preoptic area/basal forebrain is under potent inhibitory homeostatic control by corticosteroids released into the blood by the adrenal cortex. Corticosteroid levels depend on the activity of the hypothalamic–pituitary–adrenal axis, which is stimulated by activation of the serotonin system (Krueger, 2008).

Several other studies have demonstrated that differences in exposure time and IL‐1β concentration can result in very distinct effects. Short‐term exposure to IL‐1β increases perception and learning, whereas long‐term exposure reduces sensory function, impairs learning and causes fatigue (Stemkowski et al., 2015; Yadlapati & Efthimiou, 2016). Additionally, low concentrations of IL‐1β increase memory, whereas high concentrations or complete absence of IL‐1β signalling compromise memory (Goshen et al., 2007). The IL‐1R1 has been described as preferentially expressed in numerous sensory brain regions (Liu & Quan, 2018). The mechanism involved in such results is associated with the ability of IL‐1β to facilitate or inhibit the generation of long‐term potentiation, which is crucial for both learning and memory (del Rey et al., 2013). Long‐term potentiation occurs as a persistent increase of synaptic strength after high‐frequency synaptic stimulation, thus potentially coding for learning or memory processes. Interestingly, the learning process itself causes hippocampal expression of non‐inflammatory levels of IL‐1, which, in turn, helps to maintain long‐term potentiation. These results highlight the importance of a tightly regulated system.

### 4.3 Neuroendocrine functions

Interleukin‐1β induces a long‐lasting (1–2 h) corticotrophin‐releasing hormone 41 release three times higher than basal levels. Also, the intracerebral infusion of IL‐1β leads to plasma accumulation of adrenocorticotrophic hormone (ACTH) 15–20 times higher than basal levels (Barbanel et al., 1990). Similar effects were not observed after intravenous injection of IL‐1α, highlighting that although both cytokines are members of the IL‐1 family and bind to the same IL‐1R1, they might have a different spectrum of biological activities (Uehara et al., 1987). A direct effect of IL‐1β on pituitary cells has been identified as the mechanism of IL‐1β‐induced release of ACTH, luteinizing hormone, growth hormone and thyroid‐stimulating hormone (Bernton et al., 1987).

In addition, psychological stress induces IL‐1β brain expression through stimulation of the limbic–hypothalamic–pituitary–adrenal axis and secretion of ACTH and corticosterone. Interleukin‐1β stimulates the release of corticotrophin‐releasing hormone from paraventricular nuclei neurons, which can be identified by the increase in c‐fos and corticotrophin‐releasing hormone mRNA in the paraventricular nuclei neurons of the hypothalamus. Interleukin‐1β also seems to be involved in the response to stress, because it can stimulate production of the immunosuppressive glucocorticoid hormones (Gądek‐Michalska & Bugajski, 2010).

There is evidence that neurons that control the cardiovascular system use not only the classical neurotransmitters, but also non‐conventional mediators, such as angiotensin II, vasopressin, natriuretic peptides, endothelins, opioids and cytokines. Among these cytokines are IL‐1β and TNF‐α, which exert their functions by interacting with neurotransmitters or other neuromodulators (Szczepańska‐Sadowska, 2006). Interleukin‐1β plays a significant role in regulating blood pressure in resting conditions; it sensitizes brain cardiovascular neurons to the action of angiotensin II, a peptide hormone that causes vasoconstriction and increases blood pressure by acting through the angiotensin II type I receptor (Ufnal et al., 2006). In addition to resting conditions, IL‐1β intensifies the cardiovascular responses to stress (Szczepanska‐Sadowska, 2008). Also, in hypertension, IL‐1β is upregulated in two autonomic brain regions: the paraventricular nucleus of the hypothalamus and the rostral ventrolateral medulla, both of which are involved in controlling sympathetic activity and blood pressure (Elsaafien et al., 2019).

Interleukin‐1β supports processes that require high energy consumption, such as long‐term potentiation, memory and learning. Interleukin‐1β can induce its own production in the brain, which is dependent on myeloid differentiation primary response 88 activation and allows for glucoregulation. The stimulation of the metabolism in conditions of hypoglycaemia involves activation of AMPA receptors on neurons, which mediate fast synaptic transmission in the CNS, and stimulation of glucose uptake by neurons and astrocytes (Del Rey et al., 2016). Besides glucose regulation, IL‐1β suppresses food intake in rats during the first hours of the dark cycle. Similar results were observed in rats after selective vagal rootlet deafferentation and in sham‐operated rats, suggesting that subdiaphragmatic vagal afferents are not required for this phenotype (Schwartz et al., 1997). Administration of monosodium glutamate to animals causes obesity by ablation of neurons in the hypothalamic arcuate nucleus. IL‐1Ra^−/−^ mice treated with monosodium glutamate were resistant to obesity, suggesting that IL‐1β signalling antagonizes the effect of monosodium glutamate. These animals showed no weight gain, and lipid accumulation remained impaired even when fed a high‐fat diet. Both insulin levels and lipase activity were low in IL‐1Ra^−/−^ mice, which might indicate that IL‐1β is involved in lipid metabolism by regulating insulin levels and lipase activity.

## 5 CONCLUSIONS

**FIGURE 3:**
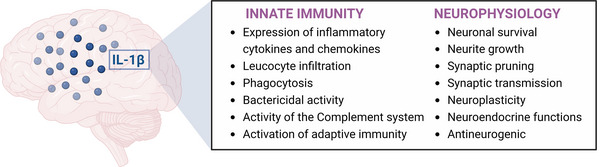
Interleukin‐1β as a key player in the CNS. Inflammatory and physiological effects of IL‐1β expression and signalling in the brain. Created with BioRender. Abbreviations: IL‐1β, interleukin‐1β.

Taken together, these findings demonstrate a pleiotropic role of IL‐1β in the brain. Depending on different conditions, such as stimuli, concentration and time of exposure, IL‐1β can act as an inflammatory mediator or a physiological mediator (Figure 3). This review has provided an overview of the mechanisms of synthesis, processing and release of IL‐1β, in addition to the signalling pathways triggered by binding of IL‐1β to its receptor (IL‐1R1). Although IL‐1β is considered primarily an inflammatory mediator, several findings point out an essential role of IL‐1β in maintaining homeostasis in several organ systems. The findings discussed here have yielded significant advances in our understanding of the physiological functions of IL‐1β in the CNS.

## AUTHOR CONTRIBUTIONS

Sole author.

## CONFLICT OF INTEREST

The author declares no conflict of interest.

## FUNDING INFORMATION

None.
